# Supplementary material for: CEN‐tools: an integrative platform to identify the contexts of essential genes
Source: Mol Syst Biol. 2020 Oct 19;16(10):e9698. doi: 10.15252/msb.20209698 (PMC7569414; doi:10.15252/msb.20209698)
Supplement: Supplementary file 1 — Appendix [file MSB-16-e9698-s001.pdf]

Appendix Figures for:

CEN-tools: An integrative platform to identify the 'contexts' of essential genes.

Sumana Sharma[1,2,#,*], Cansu Dincer[1,*], Paula Weidemüller[1], Gavin J Wright[2], Evangelia Petsalaki[1,#]

[1]European Bioinformatics Institute, Wellcome Genome Campus, Hinxton, CB10 1SD
[2]Cell Surface Signalling Laboratory, Wellcome Sanger Institute, Cambridge, UK
[3]Current address: The MRC Weatherall Institute of Molecular Medicine, Oxford, UK

[*]These authors contributed equally
[#]Correspondence should be addressed to sumana@ebi.ac.uk (Sumana Sharma) and petsalaki@ebi.ac.uk (Evangelia Petsalaki)

Contents:

Appendix Figure S1 : Project-specific discrepancy of essentiality profiles was observed for a number of genes.

Appendix Figure S2: Core essential genes have significantly higher basal expression in normal tissues than non-core genes.

Appendix Figure S3: Representative plots from the CEN-tools website for predefined contexts.

Appendix Figure S4: The co-essentiality networks of *BRAF* obtained from the PICKLES web-server.

Appendix Figure S5: The utility of CEN-tools in identification of tissue-specific gene-gene relationship.

Appendix Figure S6: CEN-tools reveals mutation dependent vulnerabilities.

Appendix Figure S7: The essentiality of FURIN in *NRAS* mutant skin cell lines is significantly higher compared to that in *NRAS* WT melanoma cell lines.

Appendix Figure S8: Examples demonstrating the utility of Cell Line Selector of CEN-tools in investigating essentiality in user defined contexts.

Appendix Figure S9: Comparison of CEN-tools core essential gene predictions with the predictions from *Dede et al*. 2020.

Appendix Figure S10: Results of applying our core essential gene prediction workflow on the "INTEGRATED" dataset from (Pacini *et al*, 2020).

Appendix Figure S11: Workflow used for the identification of core essential genes in CEN-tools.

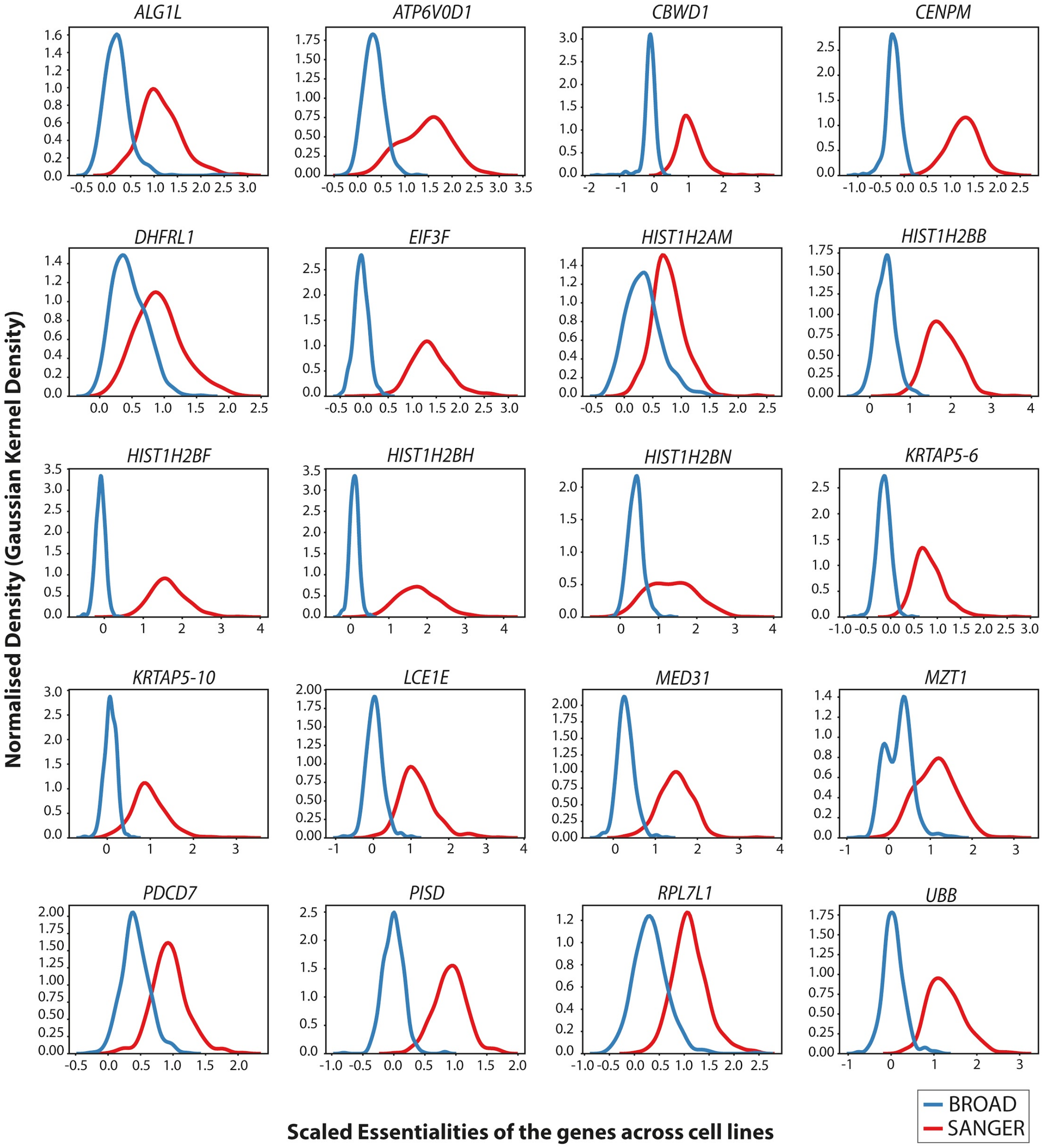

Appendix Figure S1 : Project-specific discrepancy of essentiality profiles was observed for a number of genes. Essentiality profiles of previously annotated genes from the ADaM pipeline (Behan *et al*, 2019), which were not identified as core essential genes from CEN-tools analysis pipeline. The profiles showed major discrepancies between the two projects. The ADaM pipeline utilised the essentiality screens from Project Score.

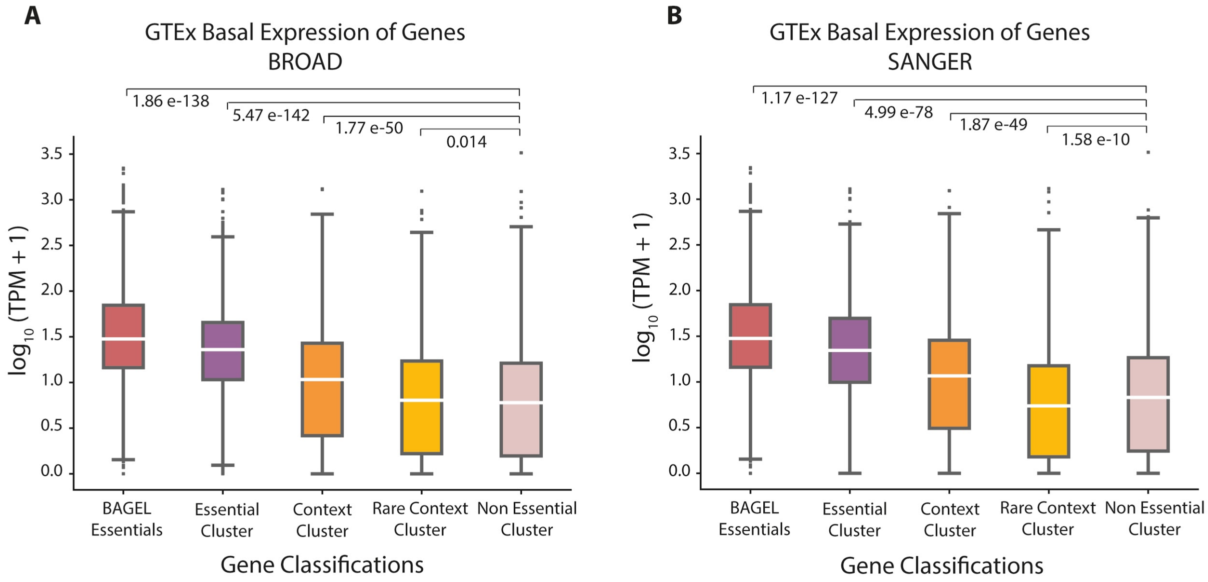

Appendix Figure S2: Core essential genes have significantly higher basal expression in normal tissues than non-core genes. Expression values were obtained from GTEx (Stranger *et al*, 2017) . Expression values in the form of $\log_{10}$ of transcript per kilobase million (TPM) with pseudocount of 1 are depicted for each of the four clusters identified from the core-analysis pipeline of CEN-tools for **(A)** BROAD project and **(B)** SANGER project. Clusters are based on essentiality probability distributions and all comparisons were performed with corresponding non essential clusters as they were the cluster with essentiality probability distribution skewed to 0. (also see **Appendix Figure S11**).

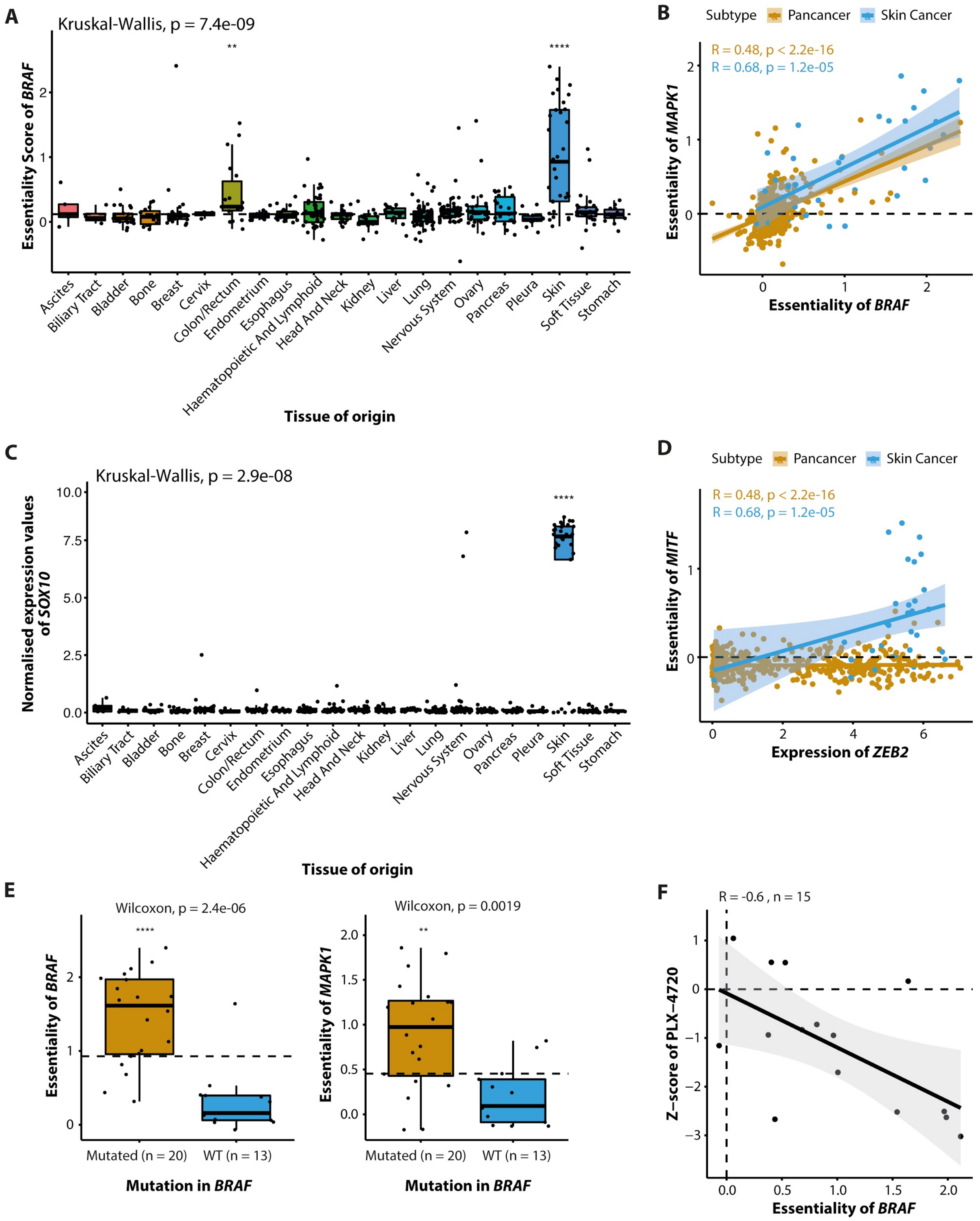

Appendix Figure S3: Representative plots from the CEN-tools website for predefined contexts. **(A)** Tissue/cancer type-wide comparisons of *BRAF* gene essentiality, **(B)** essentiality correlations of *BRAF* and *MAPK1* genes in skin tissue compared to pancancer, **(C)** tissue/cancer type-wide comparisons of *SOX10* gene expression, **(D)** correlation between essentiality of *MITF* and expression of *ZEB2* in skin tissue compared to pancancer, **(E)** essentiality of *BRAF* and *MAPK1* in skin cell lines harbouring a *BRAF* hotspot mutation (BRAFV600E) compared to skin cells with WT *BRAF* and, **(F)** correlation between drug response to PLX-4720 (*BRAF* inhibitor) and *BRAF* essentiality in melanoma cell lines. All plots show cell lines of the BROAD project.

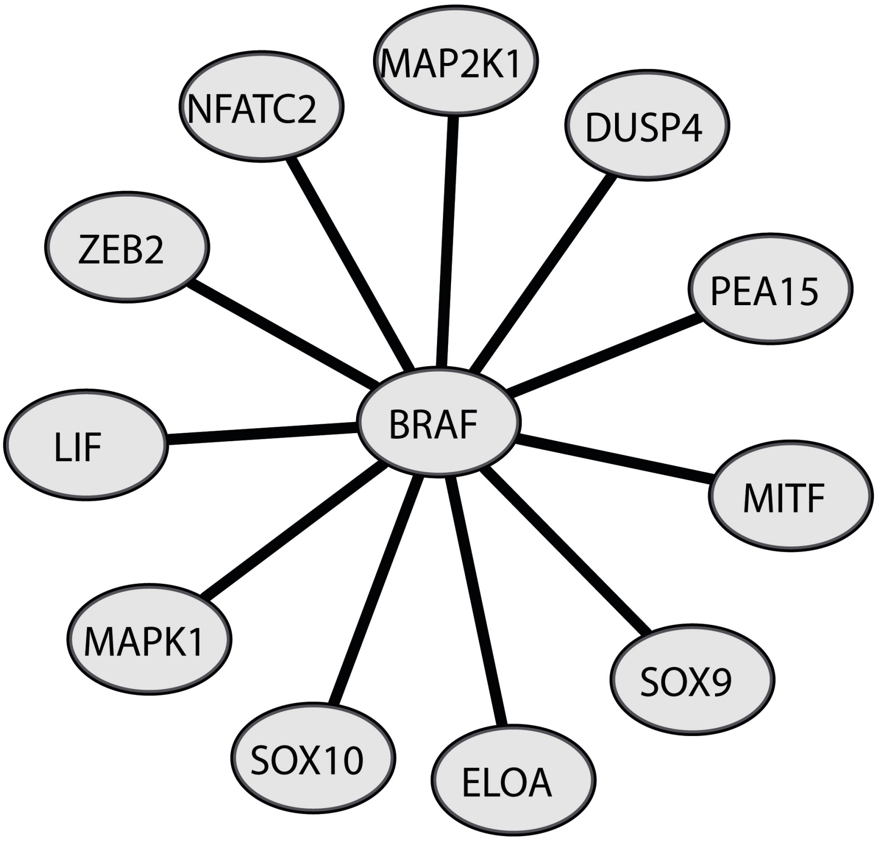

Appendix Figure S4: The co-essentiality networks of *BRAF* obtained from the PICKLES web-server. (Lenoir *et al*, 2018)

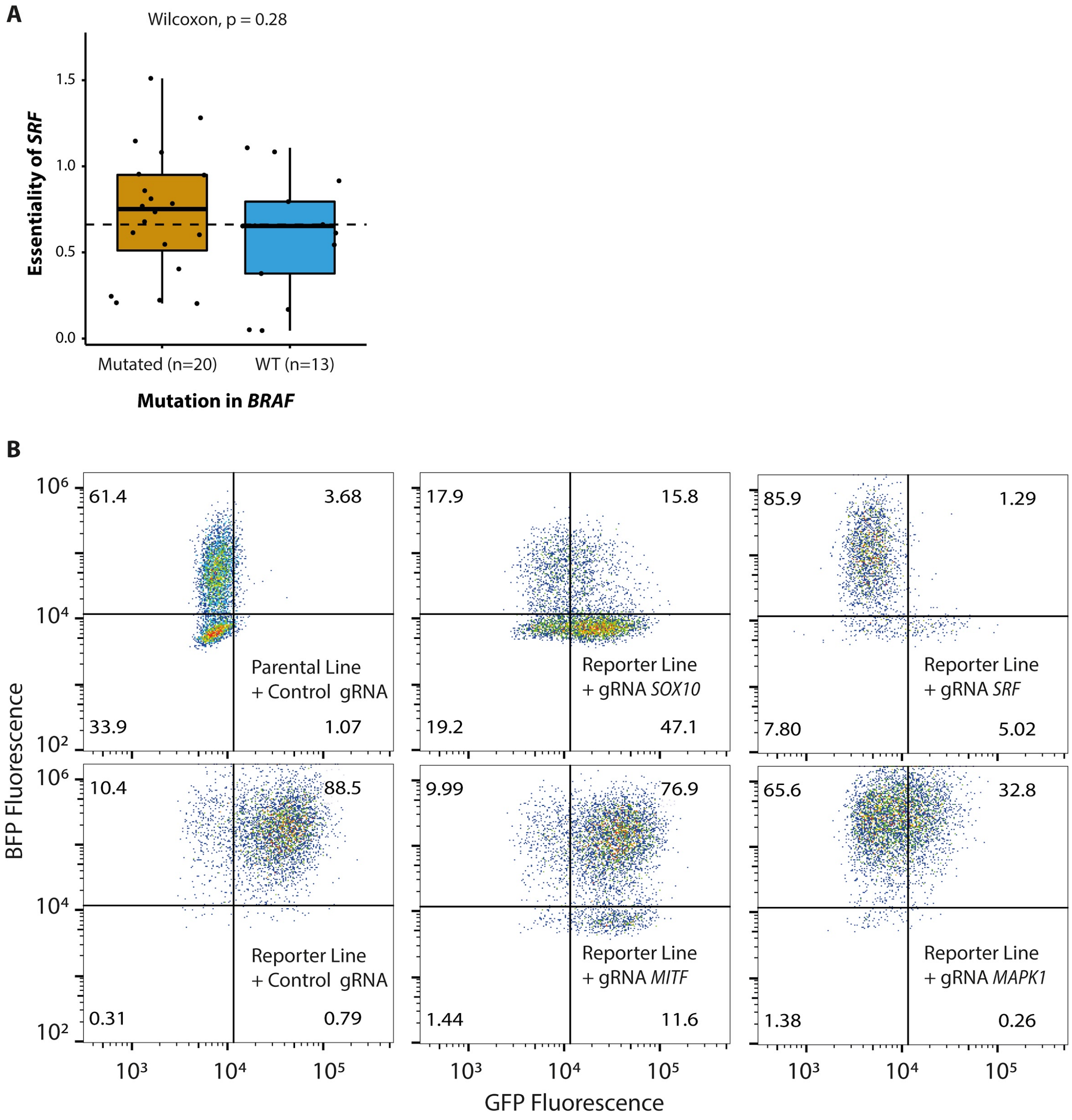

Appendix Figure S5: The utility of CEN-tools in identification of tissue-specific gene-gene relationship. (A) The essentiality of *SRF* in skin tissue is not related to the *BRAF* mutational status of the skin cancer cell lines of the BROAD project. **(B)** Representative FACS plots among 3 independent replicates depicting the effect of targeting denoted genes in the expression of GFP from the SRF-reporter construct.

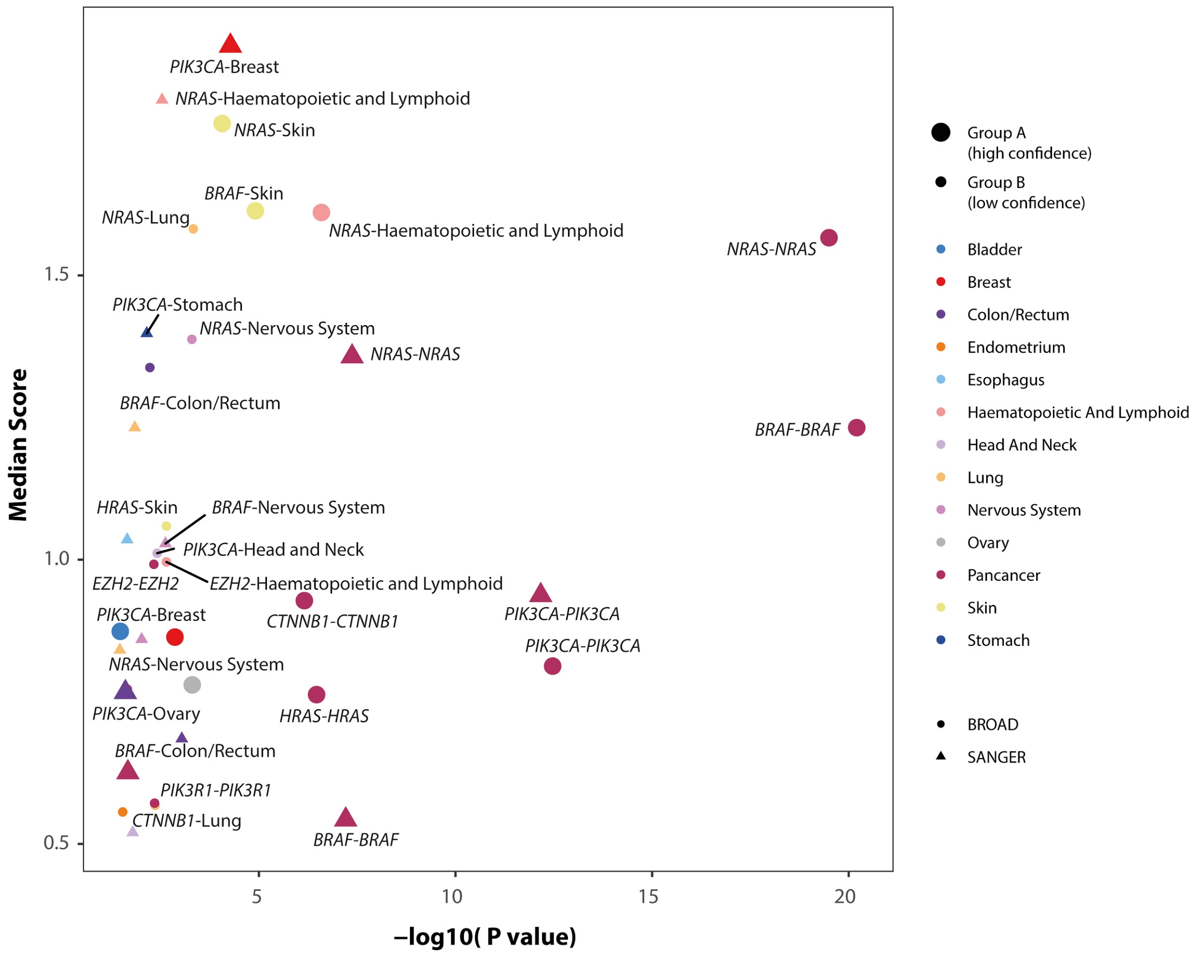

Appendix Figure S6: CEN-tools reveals mutation dependent vulnerabilities. The essentiality of oncogenes and tumor suppressor genes in the context of their own mutations in pancancer and within-tissue comparison. The 'Median Score' refers to the median of the scaled essentiality score for cell lines from the indicated project for the indicated comparison. Group A and B refer to confidence of association with Group A being higher confidence in which number of samples/group was higher than 5, compared to higher than 3 for Group B comparisons.

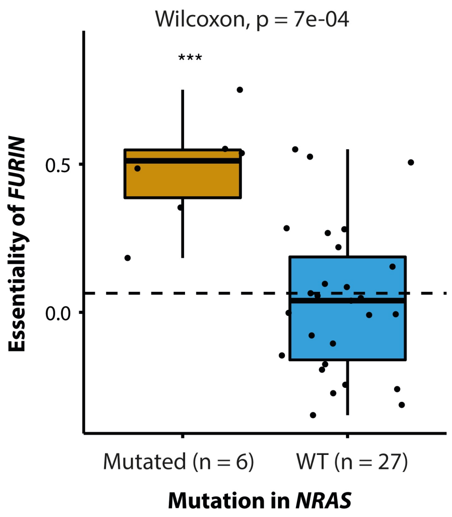

Appendix Figure S7: The essentiality of FURIN in *NRAS* mutant skin cell lines is significantly higher compared to that in *NRAS* WT melanoma cell lines. Essentiality information from 33 skin cell lines of the BROAD project were used for this analysis.

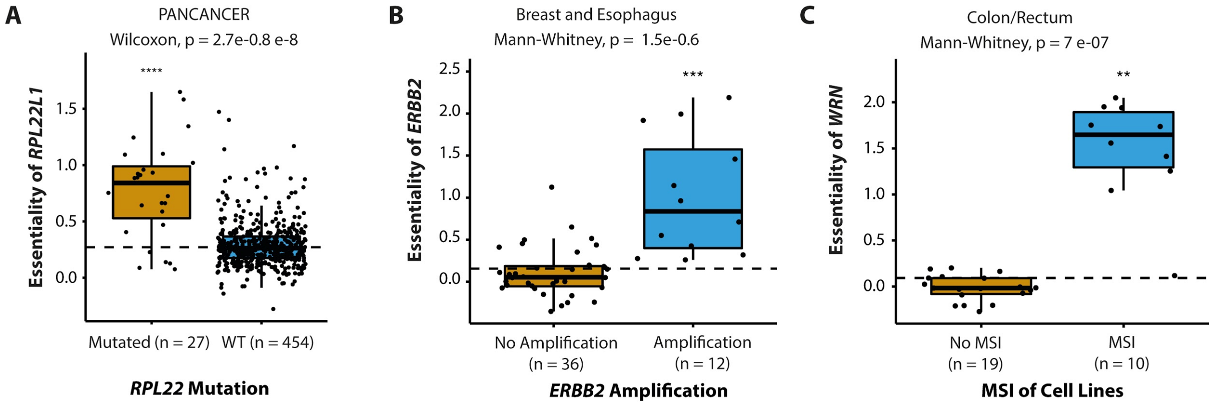

Appendix Figure S8: Examples demonstrating the utility of Cell Line Selector of CEN-tools in investigating essentiality in user defined contexts. (A) Paralog dependency: Cells with mutation in *RPL22* show dependence on paralog *RPL22L1*. **(B)** Essentiality based on CNV status: Selection of cells with amplification of *ERBB2* gene in breast and esophagus tissues reveals increased dependency on itself. **(C)** Essentiality based on microsatellite instability status (MSI): Colorectal cell lines with MSI show increased dependence on *WRN*. In all cases, cell lines in the defined context were selected using the Cell Line Selector application of CEN-tools. **(A)** and **(B)** show cell lines of the BROAD project and **(C)** shows cell lines of the SANGER project.

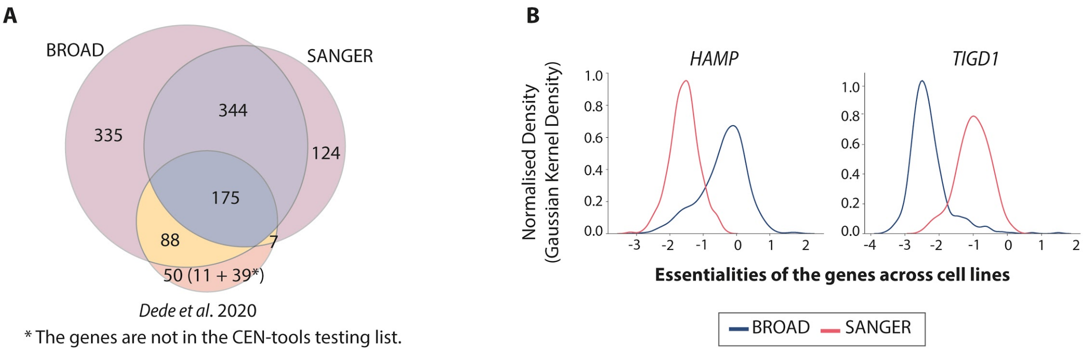

Appendix Figure S9: Comparison of CEN-tools core essential gene predictions with the predictions from *Dede et al*. 2020. (Dede *et al*, 2020) (A) Venn diagram of the core predictions from CEN-tools using both BROAD and SANGER, from *Dede et al*. Only the overlap of the newly annotated core genes from *Dede et al* are shown. 39 genes from the *Dede et al.* analysis were not in the overlapping set of genes between SANGER and BROAD, hence were not present in the testing set of CEN-tools. **(B)** Essentiality distributions of *HAMP* and *TIGD1* as representative examples of genes predicted as core-essential genes from *Dede et al.*, but not from CEN-tools. The essentiality distribution profiles of these genes show inconsistencies between the SANGER and the BROAD projects.

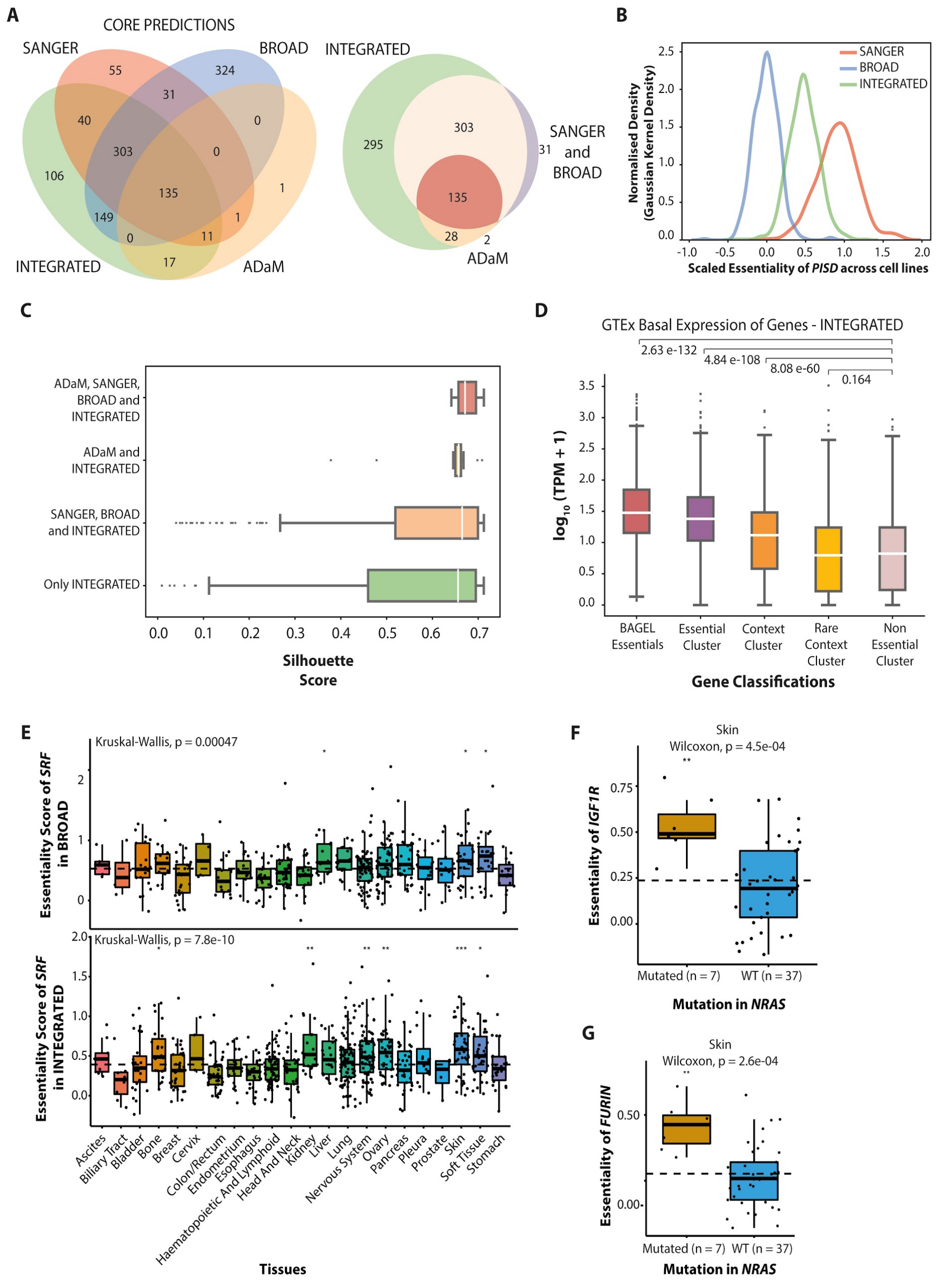

Appendix Figure S10: Results of applying our core essential gene prediction workflow on the "INTEGRATED" dataset from (Pacini *et al*, 2020). (A) Venn diagram of the core predictions from CEN-tools using INTEGRATED, SANGER and BROAD, and ADaM (Behan *et al*, 2019) using the testing gene set of INTEGRATED essentiality dataset. With the INTEGRATED, increasing the data size, the CEN-tools predictions fulfilled the ADaM core genes, except *LCE1F* and *PISD*. While *LCE1F* is not in the SANGER and BROAD gene set, *PISD* is predicted with SANGER. **(B)** Since essentiality distributions of *PISD* across all projects were not consistent with each other, *PISD* is not considered as core from CEN-tools. **(C)** Box-Plot for Silhouette Scores of core essential genes predicted via CEN-tools using BROAD, SANGER and INTEGRATED and ADaM. **(D)** Box plot for the log value of the basal expression levels from GTEx (Kundaje *et al*, 2017; GTEx Portal) of core genes in BAGEL (Hart & Moffat, 2016), ADaM, CEN-tools predictions of INTEGRATED dataset, and non-essential BAGEL and 'Not core' CEN-tools genes. **(E)** The comparison of tissue/cancer type-wide comparisons of *BRAF* gene essentiality between INTEGRATED and BROAD. Essentiality differences of **(F)** *IGF1R* and **(G)** *FURIN* with *NRAS* mutation context in skin tissue.

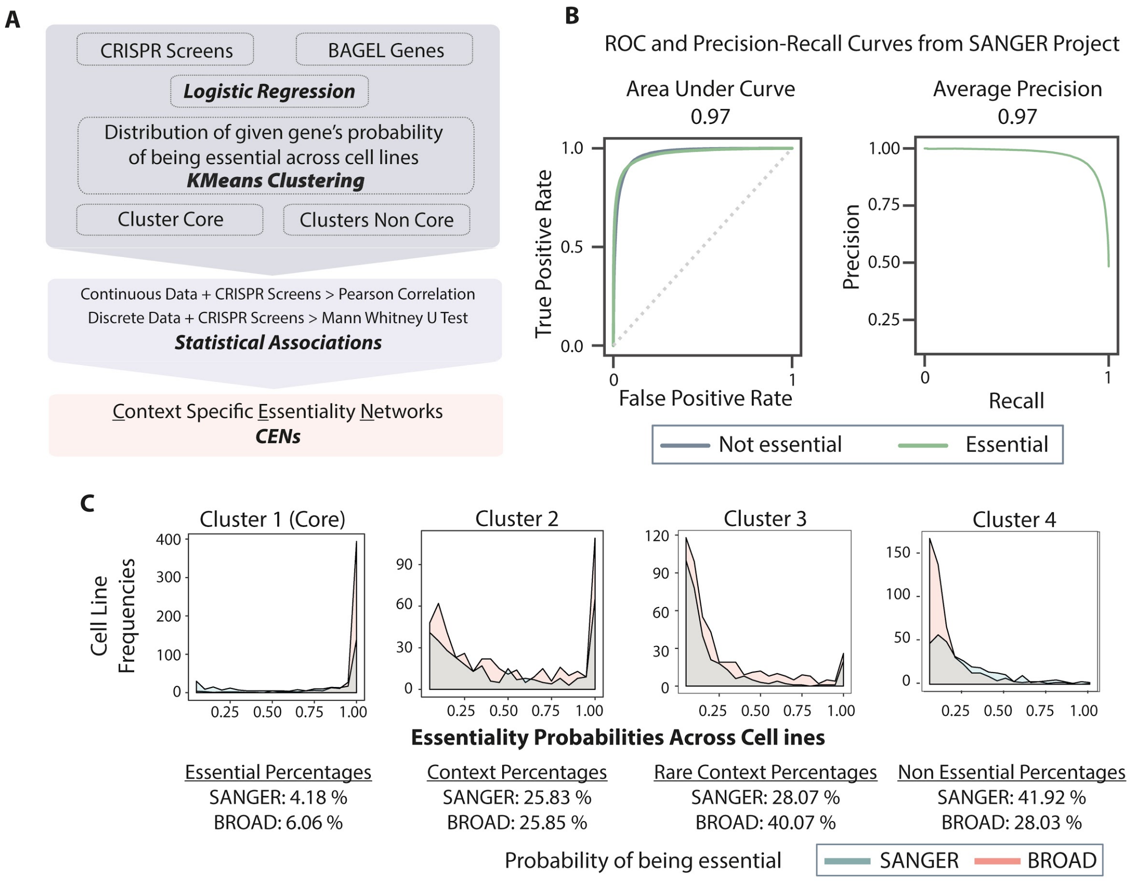

Appendix Figure S11: Workflow used for the identification of core essential genes in CEN-tools. (A) Schematic of the workflow used to create CENs. **(B)** ROC and PR curves of the Logistic Regression (LR) algorithm. **(C)** Representative essentiality probability distributions from the four different clusters depicting the probability patterns and the percentages of the number of genes in the corresponding cluster.

References

Behan FM, Iorio F, Picco G, Gonçalves E, Beaver CM, Migliardi G, Santos R, Rao Y, Sassi F, Pinnelli M, Ansari R, Harper S, Jackson DA, McRae R, Pooley R, Wilkinson P, van der Meer D, Dow D, Buser-Doepner C, Bertotti A, et al (2019) Prioritization of cancer therapeutic targets using CRISPR-Cas9 screens. *Nature* **568:** Available at: https://pubmed.ncbi.nlm.nih.gov/30971826/ [Accessed August 14, 2020]

Dede M, Kim E & Hart T (2020) Biases and Blind-Spots in Genome-Wide CRISPR Knockout Screens. *bioRxiv***:** 2020.01.16.909606 Available at: https://www.biorxiv.org/content/10.1101/2020.01.16.909606v1 [Accessed April 28, 2020]

GTEx Portal Available at: https://www.gtexportal.org/home/datasets [Accessed August 14, 2020]

Hart T & Moffat J (2016) BAGEL: a computational framework for identifying essential genes from pooled library screens. *BMC Bioinformatics* **17:** 1–7

Kundaje A, HG. Stunnenberg MH, Grundberg E, FW. Albert LK, Westra H-J, Lappalainen T, Battle A, Wright FA, Ardlie KG, O. Stegle, L. Parts, M. Piipari, J. Winn, R. Durbin, Rivas MA, Baran Y, JH. Sul, B. Han, C. Ye, T. Choi, E. Eskin, JD. Storey RT, T. Flutre, X. Wen, J. Pritchard, M. Stephens, C D Brown L M Mangravite, Das A, D Wang A Rendon, X. Wen, Y. Lee, F. Luca, R. Pique-Regi, F. Hormozdiari, E. Kostem, EY. Kang, B. Pasaniuc, E. Eskin, et al (2017) Genetic effects on gene expression across human tissues. *Nature* **550:** 204–213

Lenoir WF, Lim TL & Hart T (2018) PICKLES: the database of pooled in-vitro CRISPR knockout library essentiality screens. *Nucleic Acids Res.* **46:** D776–D780

Pacini C, Dempster JM, Gonçalves E, Najgebauer H, Karakoc E, van der Meer D, Barthorpe A, Lightfoot H, Jaaks P, McFarland JM, Garnett MJ, Tsherniak A & Iorio F (2020) Integrated cross-study datasets of genetic dependencies in cancer. **:** 2020.05.22.110247 Available at: https://www.biorxiv.org/content/10.1101/2020.05.22.110247v2.abstract [Accessed August 14, 2020]

Stranger BE, Brigham LE, Hasz R, Hunter M, Johns C, Johnson M, Kopen G, Leinweber WF, Lonsdale JT, McDonald A, Mestichelli B, Myer K, Roe B, Salvatore M, Shad S, Thomas JA, Walters G, Washington M, Wheeler J, Bridge J, et al (2017) Enhancing GTEx by bridging the gaps between genotype, gene expression, and disease. *Nat. Genet.* **49:** 1664–1670
